# Annotation of functional impact of voltage‐gated sodium channel mutations

**DOI:** 10.1002/humu.23191

**Published:** 2017-02-28

**Authors:** Valérie Hinard, Aurore Britan, Mathieu Schaeffer, Monique Zahn‐Zabal, Urs Thomet, Jean‐Sébastien Rougier, Amos Bairoch, Hugues Abriel, Pascale Gaudet

**Affiliations:** ^1^ CALIPHO GroupSIB Swiss Institute of BioinformaticsGenevaSwitzerland; ^2^ Institute of Biochemistry and Molecular MedicineBernSwitzerland; ^3^ Department of Human Protein Science, Faculty of MedicineUniversity of GenevaGenevaSwitzerland

**Keywords:** clinical interpretation of variants, databases, genetics variants, pathogenicity, phenotype, voltage‐gated sodium channel

## Abstract

Voltage‐gated sodium channels are pore‐forming transmembrane proteins that selectively allow sodium ions to flow across the plasma membrane according to the electro‐chemical gradient thus mediating the rising phase of action potentials in excitable cells and playing key roles in physiological processes such as neurotransmission, skeletal muscle contraction, heart rhythm, and pain sensation. Genetic variations in the nine human genes encoding these channels are known to cause a large range of diseases affecting the nervous and cardiac systems. Understanding the molecular effect of genetic variations is critical for elucidating the pathologic mechanisms of known variations and in predicting the effect of newly discovered ones. To this end, we have created a Web‐based tool, the Ion Channels Variants Portal, which compiles all variants characterized functionally in the human sodium channel genes. This portal describes 672 variants each associated with at least one molecular or clinical phenotypic impact, for a total of 4,658 observations extracted from 264 different research articles. These data were captured as structured annotations using standardized vocabularies and ontologies, such as the Gene Ontology and the Ion Channel ElectroPhysiology Ontology. All these data are available to the scientific community via neXtProt at https://www.nextprot.org/portals/navmut.

## 1 INTRODUCTION

**Figure 1:**
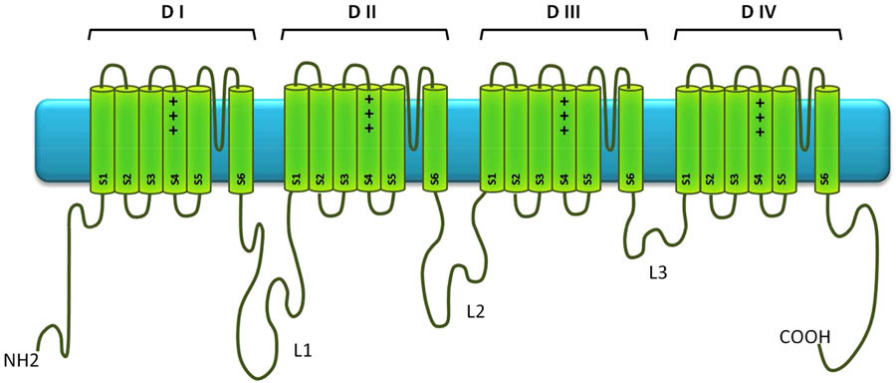
Topology of voltage‐gated sodium channels

Ion channels are integral membrane proteins that allow ions to flow across membranes in all living cells, playing an important role in key physiological processes such as neurotransmission, muscle contraction, learning and memory, secretion, cell proliferation, regulation of blood pressure, fertilization, and cell death. In humans, 344 genes encode the pore‐forming subunits of ion channels. Mutations in more than 126 ion channel as well as in several ion channel‐interacting protein genes have been reported to cause diseases, known as channelopathies (Ashcroft, 2006). The voltage‐gated sodium channel group comprises nine members in mammals: *SCN1A*–*SCN5A*, corresponding to Na_v_1.1 to Na_v_1.5, and *SCN8A*–*SCN11A*, corresponding to Na_v_1.6 to Na_v_1.9 (Table 1). *SCN7A* corresponding to Na_x_ is not included in this group as it is not voltage sensitive. These channels are proteins of about 2,000 amino acids that are composed of four domains (DI–DIV), each consisting of six transmembrane helices (S1–S6) and connected to each other by the L1, L2, and L3 linkers (Figure 1). They interact with other proteins that modulate channel gating properties as well as channel trafficking and subcellular localization. Regions known to be involved in the gating properties and the protein–protein interactions are listed in Table 2 (Ahern, Payandeh, Bosmans, & Chanda, 2016).

**Table 1:** Diseases associated with mutations in voltage‐gated sodium channels

Gene	Protein	OMIM ID	neXtProt ID	Major Expression	Human Disorders due to Mutations in Voltage‐Gated Sodium Channels
*SCN1A*	Nav1.1	182389	NX_P35498	CNS, PNS	Generalized Epilepsy with Febrile Seizures Plus, Dravet Syndrome, Febrile Seizure, Familial Hemiplegic Migraine, Acute Encephalopathy, Juvenile Myoclonic Epilepsy Intractable without Status Epilepticus
*SCN2A*	Nav1.2	182390	NX_Q99250	CNS, PNS	Epileptic encephalopathy, Dravet Syndrome, Autism, Intellectual Disability, Generalized Epilepsy with Febrile Seizures Plus
*SCN3A*	Nav1.3	182391	NX_Q9NY46	CNS, PNS	Partial Epilepsy, Generalized Epilepsy with Febrile Seizures Plus
*SCN4A*	Nav1.4	603967	NX_P35499	Skeletal muscle	Hypokalemic Periodic Paralysis, Hyperkalemic Periodic Paralysis, Paramyotonia Congenita, Myotonic Disorder
*SCN5A*	Nav1.5	600163	NX_Q14524	Cardiac muscle	Brugada Syndrome, Long QT Syndrome, Ventricular Arrhythmia, Sudden Infant Death Syndrome, Dilated Cardiomyopathy, Sick Sinus Syndrome
*SCN8A*	Nav1.6	600702	NX_Q9UQD0	CNS, PNS	Epileptic encephalopathy, Migrating Partial Seizures in Infancy, Intellectual Disability
*SCN9A*	Nav1.7	603415	NX_Q15858	PNS	Primary Erythromelalgia, Pain Insensitivity, Paroxysmal Extreme Pain Disorder, Small Fiber Neuropathy
*SCN10A*	Nav1.8	604427	NX_Q9Y5Y9	PNS	Peripheral Neuropathy, Primary Erythromelalgia, Small Fiber Neuropathy
*SCN11A*	Nav1.9	604385	NX_Q9UI33	PNS	Small Fiber Neuropathy, Episodic Pain Syndrome Familial 3, Neuropathy Hereditary Sensory and Autonomic Type VII

*Note*: CNS, central nervous system; PNS, peripheral nervous system.

**Table 2:** Regions related to sodium channel's function

Channel Function	Interaction Partners	Region
**Voltage sensing domain**		S1, S2, S3, and S4
**Filter pore**		S5‐S6 loop
**Inactivation gate**		L3
**Site of inactivation gate**		S4‐S5 loop in DIII
	**Beta partners**	N‐term and C‐term
	**Kinases**	
	PKC	L1 and L3
	PKA	L1
	CSNK2A2	L2
	GSK3B	C‐term
	**Trafficking partners**	
	Ankyrin	L2
	Calmodulin	C‐term
	NEDD4L	C‐term
	FGF	C‐term

Disruption of any aspect of the channel function can cause a wide spectrum of diseases. Depending on their tissue expression, defects in their function cause disorders such as epilepsy and seizures, paralysis, myotonia, pain disorders, autism, cognitive impairments, as well as many cardiac arrhythmic syndromes (Table 1). Several genetic variations are known to affect voltage‐gated sodium channels leading to a defect in their cellular or biophysical properties, and consequently to the development of diseases. Knowledge on the effect of these mutations is spread across the scientific literature. Consolidating these data in a single platform will help scientists and clinicians to have a source of validated information for general reference, as well as to help in clinical applications such as diagnosis, genetic, and therapeutic counseling.

Undeniably, there is a need for an integrated and comprehensive resource that describes the current state of knowledge of the impact of mutations at the molecular and/or cellular levels, in a format compatible with computational analysis. Some clinically relevant variants are reported in several public databases, such as ClinVar (https://www.ncbi.nlm.nih.gov/clinvar/) and OMIM (http://omim.org/). These resources mainly focus on the clinical aspects of variants. Channelpedia (http://channelpedia.epfl.ch/), developed by the Blue Brain Project, was created with the intention of capturing all information related to ion channels. However, its content is specifically focused on data required for biophysical modeling and has limited information on the biological roles and molecular function of individual protein residues. Another pertinent resource is IUPHAR‐DB (http://www.guidetopharmacology.org/), which is developed by the International Union of Basic and Clinical Pharmacology. This database includes genetic, proteomic, pharmacological, some functional, and biophysical characteristics but lacks detailed information on the functional impact of sequence variations. Finally, there are also several locus‐ or disease‐specific resources that exist such as http://www.molgen.vib-ua.be/scn1amutations/; http://www.gzneurosci.com/scn1adatabase/; https://gene.sfari.org/GeneDetail/SCN2A; http://triad.fsm.it/cardmoc/ (last modified in 2010). However, none of the above resources is provided in a format that is amenable to computational analyses and many of them have not been updated for years.

In this article, we describe the generation of the neXtProt Ion Channels Variants Portal, available at https://www.nextprot.org/portals/navmut, a corpus of data of voltage‐gated sodium channel mutations consisting of over 4,600 observations at the molecular and/or cellular level on 672 variants. The unique features of the portal are as follows: we captured both natural mutations found in patients, as well as artificial mutations designed to study specific aspects of protein function in vitro and/or in vivo; we annotated the phenotypic impact of variants for all nine members of the voltage‐gated sodium channel family; all data are represented using standardized vocabularies; and last but not least, each statement is reviewed by an expert curator and goes through a thorough quality control process.

## 2 MATERIALS AND METHODS

### 2.1 Selection of data for curation

The Ion Channels Variants Portal presents data on the molecular, cellular, and organ‐level phenotypes caused by genetic variations in the nine human voltage‐gated sodium channels. Only variations that affect the coding sequence and give a defined mutated product have been considered, excluding intronic or splice site variations. This information was manually extracted from research articles indexed in PubMed and captured using an in‐house developed biocuration software platform, the BioEditor. This tool allows the capture of structured annotations using standardized vocabularies. The experimental evidence supporting each annotation is also captured, including the reference, the type of assay, the protein origin, and the biological system in which the experiment was performed.

### 2.2 Data model

Annotation statements (Table 3A) are triplets composed of (1) a subject that corresponds to the protein variation being annotated; (2) an object that describes the function being tested; (3) a relation describing how the function of the protein is affected by the mutation.

**Table 3:** Annotation elements: **(A)** basic triplet statement for “SCN5A‐p.Ser2014*” variant, **(B)** relations, and **(C)** evidence for the triplet “SCN5A‐p.Ser2014* decreases macroscopic conductance”

A. ANNOTATION		
Element	CV/Ontology	Example
Subject	HGVS nomenclature	SCN5A‐p.Ser2014*
Relation	cv_modification_effect.obo	Decreases
Object	BioObject/GO/ICEPO/ Protein Property/MGI phenotype	Binding to SNT (BioObject) cardiac conduction (GO) macroscopic conductance (ICEPO) protein abundance (Protein Property)

B. RELATIONS	
**No impact**	No significant effect observed compared with the wild‐type
**Does not cause phenotype**	No observable morphological, physiological, and behavioral characteristics in the mutant compared with the wild‐type
**Impacts**	Some significant effect observed compared with the wild‐type
**Causes phenotype**	Some observable morphological, physiological, and behavioral characteristics in the mutant compared with the wild‐type
**Increases**	Some significant increase observed in a quantifiable measure compared with the wild‐type
**Decreases**	Some significant decrease observed in a quantifiable measure compared with the wild‐type
**Gains function**	Mutant protein acquires a property absent from the wild‐type (new substrate, new cellular localization, etc.)
**Depolarizes**	Some significant shift of the curve in the depolarizing direction compared with the wild‐type
**Hyperpolarizes**	Some significant shift of the curve in the hyperpolarizing direction compared with the wild‐type
**Hastens**	Some significant higher value in the transition speed compared with the wild‐type
**Slows**	Some significant lower value in the transition speed compared with the wild‐type
**Impacts temperature‐dependence of**	Some significant impact dependent on the temperature compared with the wild‐type

C. EVIDENCE		
Element	CV/Ontology	Example
Evidence code(s)	Evidence and conclusion ontology	Whole‐cell voltage clamp recording protocol/knockin evidence used in manual assertion/current density
Protein origin	NCBI taxonomy	Mus musculus (NCBITaxID10090)
Biological model species, cell type, anatomy, cell line	NCBI taxonomy CALOHA anatomy ontology Cellosaurus	Mus musculus (NCBITaxID10090) Heart muscle cell (CALOHA:TS‐0115)
Phenotype intensity	Mild/moderate/severe	Moderate
Evidence quality	Gold/silver	Gold
Reference	Cross‐reference to PubMed	PUBMED 24895455

The *subjects* are protein variations described using the HGVS nomenclature (den Dunnen, Dalgleish et al., 2016). The origin of the variation, either germline, somatic, or generated by site‐directed mutagenesis, as well as its consequence on the protein (non‐synonymous codon, stop gained, frameshift variant, in‐frame codon gain, and in‐frame codon loss), are captured. The positions of the variations on the protein sequence refer to the respective neXtProt entries (for example *SCN1A* corresponds to NX_P35498 and *SCN9A*‐iso3 to NX_Q15858‐3 in https://www.nextprot.org/).

The *objects* represent either (1) molecular and biological processes or localizations from the Gene Ontology (GO) vocabulary (The Gene Ontology Consortium, 2015), (2) electrophysiological parameters described with the Ion Channel ElectroPhysiology Ontology (ICEPO), a specific ontology developed in our group (Hinard et al., 2016), (3) mammalian phenotypes from the Mammalian Phenotype Ontology (Smith & Eppig, 2012), (4) proteins, represented by Gaudet et al. 2017 to describe effects on protein–protein interactions, or (5) protein property, such as protein abundance and stability, represented by an in‐house developed protein property vocabulary (ftp://ftp.nextprot.org/pub/current_release/controlled_vocabularies/cv_protein_property.obo).

The *relations* (Table 3B) linking the subject and the object are grouped into two main concepts: “no impact” and having “impact.” The possible impacts of variations can be further specified using “increases,” “decreases,” or “gains function”; “causes phenotype” for mammalian phenotype terms and specific impacts of variations on electrophysiological parameters can be described with relevant relations such as “depolarizes,”, ”hyperpolarizes,” “hastens,” “slows,” or “impacts temperature‐dependence of.”

For each annotation, detailed information about the experimental support of each statement is captured as evidence statements (Table 3C). The annotation evidence is composed of (1) one or more terms from the Evidence and Conclusion Ontology (Chibucos et al., 2014), describing the experiment performed, such as basic biological experiments or specific types of electrophysiological recordings; (2) the protein origin, which represents the species from which the protein was obtained for the experiment described using the NCBI taxonomy (https://www.ncbi.nlm.nih.gov/taxonomy); (3) the biological system in which the experiment was done, that may contain one or more of these elements: the organism from the NCBI taxonomy, the tissue or cell type, from the CALOHA human anatomy vocabulary (ftp://ftp.nextprot.org/pub/current_release/controlled_vocabularies/caloha.obo) or the cell line from the Cellosaurus knowledge resource (http://web.expasy.org/cellosaurus/); (4) a qualitative assessment of the severity of the phenotype, either “Mild,” “Moderate,” or “Severe”; (5) a quality flag; each evidence is labeled as either Gold (high quality) or Silver (good quality), knowing that the "Silver" tag is used where quality of the experiment is not optimal according to curator's judgment; and (6) one reference, usually identified using a PubMed ID.

### 2.3 Assessment criteria for phenotype severity and data confidence

The criteria used to grade the severity of the phenotypic observations are based on the fold‐change of activity, response, and so on, in the mutant compared with the wild‐type control under the same conditions. A phenotype is considered “Mild” when the change is around 10%–20% of the control. “Moderate” is assigned when the change is between 20% and 80% of the control, whereas “Severe” is used to describe changes exceeding 80% of the control.

Each evidence is also assigned a qualitative confidence score, Gold or Silver. This is subjective to some extent and varies on a case‐by‐case basis, but our guidelines for assigning Gold quality to an experiment require either good statistical significance (*P*< = 0.01; for experiments where appropriate) or substantial relevance of the experiment, including appropriate controls. For example, a Silver tag may be assigned when the data are qualitative and/or statistical evaluation is missing, when errors are very large, when the data result from a low confidence assay (for example, low replicate number; poorly defined experimental systems, etc.), or when the experiment is carried out in a non‐human protein that is evolutionarily distant from the human protein.

### 2.4 Quality control

To ensure data integrity, the annotations undergo both automated and manual checks. Automated checks ensure that the annotation is complete, that is, it contains a subject, a relation, an object, a reference, at least one evidence code, and the species in which the experiment was done. In the case of sequence variations, our software checks that the original amino acid at the position annotated is found in the sequence being annotated. Also, manual checks are performed to provide correct and consistent annotations. For example, controlled vocabulary terms used in the annotations are checked to ensure that terms used for biological processes were consistent throughout the annotations corpus. Once all the checks are successful, the annotations obtain a “Valid” status and are integrated into the portal.

## 3 RESULTS AND DISCUSSION

### 3.1 Creation of the Ion Channels Variant Portal

**Figure 2:**
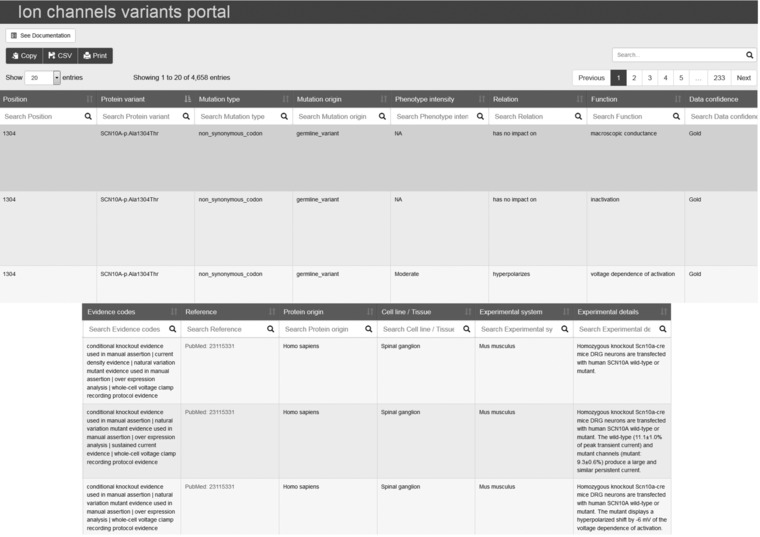
Screenshot of the Ion Channels Variants Portal. Each column can be filtered for specific values and sorted alphabetically or numerically, depending on the data type. The number of entries corresponding to a search result appears above the table

The Ion Channels Variants Portal is specific to voltage‐gated sodium channel gene family (SCN). It contains all pertinent knowledge concerning mutations in the nine human voltage‐sensitive sodium channel proteins and their impact on the biophysical properties and the biological processes of the channels. The portal provides an intuitive tool for rapidly searching and comparing the functional information on Na_v_ variants. All this information is publicly available at https://www.nextprot.org/portals/navmut. The data are presented in table format, as shown in Figure 2. The data are ordered alphabetically by gene name and then by variant position. In addition, each column can be filtered for specific values and the selected data can be downloaded in comma‐separated or PDF format for a deeper analysis. The content of each column is described in the portal documentation (“See documentation” link).

### 3.2 Impact of Na_v_ variants on protein function

The Ion Channels Variants Portal contains 4,658 phenotypic observations on 672 variants, both natural and artificially generated with at least one phenotypic impact, extracted from 264 publications (Table 4). The corpus of data available for each Na_v_ varies widely, with *SCN2A*, *SCN4A*, and *SCN5A* having the highest number of variants, whereas *SCN3A* and *SCN11A* have the fewest number of variants. *SCN1A*, *SCN8A*, *SCN9A*, and *SCN10A* have nearly identical number of variants and an intermediate number of annotations compared with the others. Most of the molecular and cellular assays done on the sodium channels target electrophysiological parameters: about 80% of the assays reported in the literature are relevant to channel electrophysiological functions, whereas only 20% of the experiments test the sub‐cellular localization, interactions, stability, and so on of the channels.

**Table 4:** Number of variants and annotations in the Ion Channels Variants Portal per channel and number of disease‐associated variants obtained from the ClinVar and neXtProt databases. The last row presents the proportion of naturally occurring variants from our set of variants for each channel

	SCN1A	SCN2A	SCN3A	SCN4A	SCN5A	SCN8A	SCN9A	SCN10A	SCN11A	Total
**Number of observations**	535	860	51	1,294	1,063	163	514	119	59	**4,658**
**Number of variants**	51	184	7	200	112	27	59	27	5	**672**
**Number of disease‐associated variants**	506	50	6	72	529	26	62	27	11	**1,289**
**Proportion of natural variants**	**96%**	**12%**	**86%**	**20%**	**82%**	**22%**	**75%**	**27%**	**100%**	

### 3.3 Distribution of Na_v_ variants in different topological regions, domains, and segments

To simplify the presentation of the variants distribution, we first divided the proteins based on two topological types: the cytoplasmic regions composed of the amino‐ and carboxyl‐termini as well as the three linkers L1, L2, and L3, and the DI–DIV domains (mostly composed of transmembrane regions).

**Figure 3:**
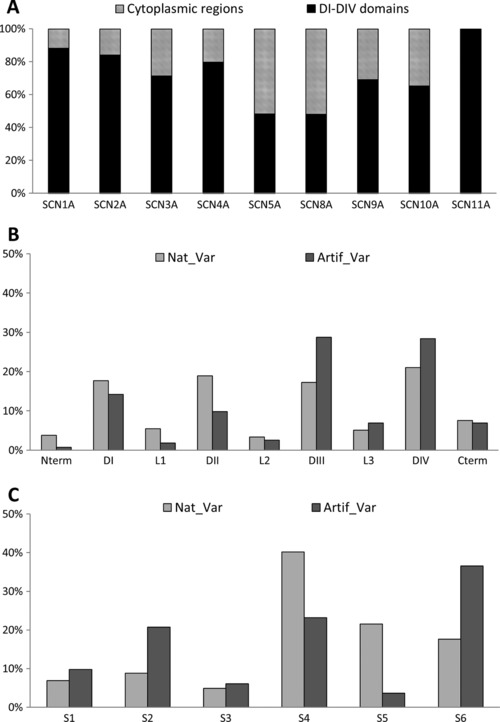
Distribution of the variants according to the topological domains: **A**: DI–DIV domains versus cytoplasmic regions. Distribution of the variants with electrophysiological defects: the natural variants (Nat_Var) versus artificial variants (Artif_Var) according to (**B**) the domains or to (**C**) the segments

Consistent with the important role of the DI–DIV domains in channel gating (Bennett et al., 1995), most variants are located in these regions (Figure 3A): seven out the nine channels have over 60% of their variants in the DI–DIV domains. However, the *SCN5A* and *SCN8A* variants are evenly distributed between the domains and cytoplasmic regions. In the case of *SCN5A*, the cytoplasmic regions have been extensively studied because they are targeted by a high proportion of disease‐causing mutations. The underlying defect of the *SCN5A* mutations in the L3 and the C‐terminal regions has been linked to their role in the inactivation gating process (Bennett et al., 1995; Mantegazza, Yu, Catterall, & Scheuer, 2001; Wehrens et al., 2003). It is not clear why *SCN8A* has a smaller fraction of mutations in the DI–DIV domains compared with other Na_v_s; it may be a bias due to the relatively small number of variants analyzed.

Another interesting point is to see whether there is a difference in the localization of the variants with respect to their origin (natural versus artificial). From the 672 characterized variants, 272 are natural and 400 are artificial (40% and 60%, respectively). Analysis of their distribution reveals that there are a majority of artificially generated variants in the DI–DIV domains (Table 5). While the artificial variants are generated primarily to understand channel function, with an emphasis on the important DI–DIV domains, the same regions are targeted by disease‐associated variants. Therefore, the inclusion of artificially generated variants in the annotation corpus does not bias the data analysis but on the contrary contributes to the understanding of variation impacts.

**Table 5:** Number of natural (Nat_var) or artificial variants (Artif_var) present in DI–DIV domains or cytoplasmic regions (cyto)

	SCN1A	SCN2A	SCN3A	SCN4A	SCN5A	SCN8A	SCN9A	SCN10A	SCN11A	Total
**Nat_var in DI–DIV domains**	43	17	6	30	47	5	33	5	5	**191**
**Nat_var in cyto regions**	6	6	0	10	45	1	11	2	0	**81**
**Artif_var in DI–DIV domains**	2	137	0	130	7	8	8	12	0	**304**
**Artif_var in cyto regions**	0	24	1	30	13	13	7	8	0	**96**
**Total of variants**	**51**	**184**	**7**	**200**	**112**	**27**	**59**	**27**	**5**	**672**

### 3.4 Distribution of electrophysiological phenotypes

#### 3.4.1 Distribution of variants within DI**–**DIV domains

SCN proteins are composed of four domains (DI–DIV; Figure 1), each made up of six membrane spanning helical segments (S1–S6). The four domains of Na_v_ channels form the central ion conducting pore and the voltage‐sensor domain (VSD) of the protein. The DI–DIII domains are essentially involved in activation, whereas the DIV plays a specific role in fast inactivation (Ahern et al., 2016; Goldschen‐Ohm, Capes, Oelstrom, & Chanda, 2013). Variations impacting the electrophysiological parameters are mostly found in these four domains. The proportion of natural and artificial variants are roughly similar for all regions, except for domains DIII and DIV that contain more artificial mutants due to their extensive study in *SCN2A* and *SCN4A* (Figure 3B).

#### 3.4.2 Distribution of variants within S1–S6 transmembrane segments

Looking in more detail at the S segments regardless of the domain they belong to, we observed that the natural variants impacting electrophysiological parameters are distributed predominantly in the S4 segment (41%), whereas the artificial variants are mainly localized in the S6 segment (37%) (Figure 3C). The S6 segments are critical for permeation and fast inactivation because of their close proximity with the selectivity filter and inactivation gate (McPhee, Ragsdale, Scheuer, & Catterall, 1994, 1995), whereas the S4 segments play an essential role in VSD (Stuhmer et al., 1989). In addition, the S4 segments of the DIV domain have been shown to play a unique role in activation–inactivation coupling (Chen, Santarelli, Horn, & Kallen, 1996). Interestingly, a large fraction of disease‐causing variants are also localized in these segments: 25% in S4 and 23% in S6. Thus as expected, the electrophysiological variant distribution correlates with the importance of the D domains and the S segments on the channel function.

### 3.5 Distribution of non‐electrophysiological phenotypes

Finally, we looked at the annotations affecting phenotypes other than electrophysiological parameters such as the binding, signaling, effect on cellular function, localization, and degradation. We noticed that these variants are mostly localized in the cytoplasmic regions (N‐terminus, linkers, and C‐terminus) rather than in the domains DI–DIV (63% and 37%, respectively) that is most likely related to the fact that transmembrane sequences from the domains offer less physical access to binding. All these variants impair interactions with intracellular proteins (outlined in Table 2) that regulate the trafficking and/or activity of the channels, including protein kinases (PKC, PKA, *CSNK2A2*, *GSK3B*, etc.), ubiquitin‐protein ligases (*NEDD4* and *NEDD4L*), and trafficking partners such as Ankyrin (*ANK2* and *ANK3*), Syntrophin (*SNTA1*, *SNTB1*, *SNTB2*, *SNTG1*, *SNTG2*), fibroblast growth factor homologous factors 1–4 (*FGF‐11/‐12/‐13/‐14*) calmodulin, and so on (Abriel & Kass, 2005).

### 3.6 Comparison of disease‐associated variants and experimentally characterized variants

**Figure 4:**
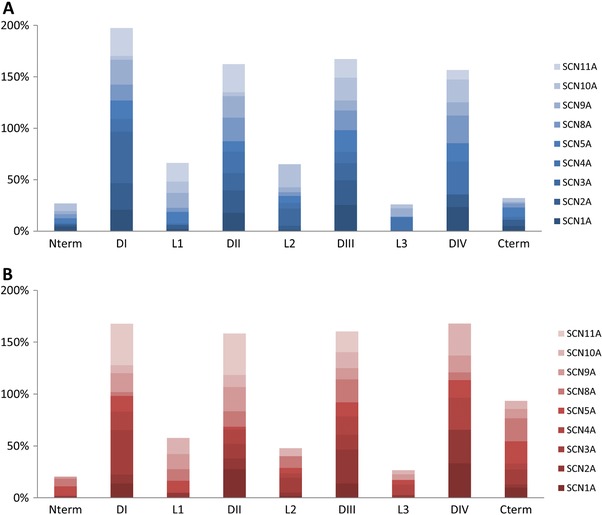
Stacked fraction of (**A**) the disease‐causing variants per region and of (**B**) the characterized variants per region

One important challenge in medicine is to correlate the symptoms of patients (phenotypes) with the causative genetic variations. Conversely, as genome sequencing is expected to become a standard medical laboratory practice, it may be possible to identify mutations in individuals before any symptoms are visible. This practice may be especially relevant for Na_v_ variants, as several associated diseases have a relatively late onset and mild disease progression, thus providing ample opportunities for disease prevention or attenuation if diagnosed in time. Our portal has therefore important potential applications in this area. A total of 1,289 disease‐associated variants in the nine voltage‐gated sodium channels were obtained from ClinVar and neXtProt (Table 4), 18% of which the portal has functional annotations (231 variants). Hence, the largest fraction of disease‐associated variants is not characterized. Nevertheless, there are 441 variants not described to cause diseases but with functional data that could contribute to understand the pathogenic potential of new or paralogous variants. A detailed view of the overlap in the disease variants and the variants characterized experimentally is shown in Figure 4 where the fraction of variants in each of the domains and linkers is shown for each Na_v_. The overall distribution of the variants with phenotypic data generally matches that of the disease variants, except for the C‐terminal region. This exception is likely due to the specific research focus on the regulatory interactions occurring in this region, for example with calmodulin and NEDD4L. These data indicate that despite the importance of this C‐terminal region, only few natural mutations have been found in this region and shown to trigger disorders.

## 4 CONCLUSIONS

The Ion Channels Variants Portal we have developed provides an exhaustive list of variants in voltage‐gated sodium ion channels for which molecular phenotypes are available, curated in a highly structured model, with detailed information about the experimental system, without redundancy in the data and with complete traceability to the original experimental results. Researchers as well as clinical geneticists will be able to consult this database to have a comprehensive overview of the available data, which may be used to support the clinical decision process. Furthermore, with the large amount of data available, correlations between different mutations and certain diseases may be used to predict the effect of similar mutations in paralogous proteins.

The Ion Channels Variants Portal will undoubtedly be a useful resource for a better understanding of ion channel function, essential for understanding channelopathies. To be consistent with this aim, we will continue our effort to integrate newly characterized Na_v_ variants and we welcome contact with groups having data sets that they would like to be considered for inclusion in the portal. Finally, we also plan to use the same approach to expand the annotation corpus to other ion channels.
